# Subcellular Location, Phosphorylation and Assembly into the Motor Complex of GAP45 during *Plasmodium falciparum* Schizont Development

**DOI:** 10.1371/journal.pone.0033845

**Published:** 2012-03-30

**Authors:** Mohd A. Mohd Ridzuan, Robert W. Moon, Ellen Knuepfer, Sally Black, Anthony A. Holder, Judith L. Green

**Affiliations:** 1 Division of Parasitology, MRC National Institute for Medical Research, London, United Kingdom; 2 Herbal Medicine Research Center, Institute for Medical Research, Jalan Pahang, Kuala Lumpur, Malaysia; Institut national de la santé et de la recherche médicale - Institut Cochin, France

## Abstract

An actomyosin motor complex assembled below the parasite's plasma membrane drives erythrocyte invasion by *Plasmodium falciparum* merozoites. The complex is comprised of several proteins including myosin (MyoA), myosin tail domain interacting protein (MTIP) and glideosome associated proteins (GAP) 45 and 50, and is anchored on the inner membrane complex (IMC), which underlies the plasmalemma. A ternary complex of MyoA, MTIP and GAP45 is formed that then associates with GAP50. We show that full length GAP45 labelled internally with GFP is assembled into the motor complex and transported to the developing IMC in early schizogony, where it accumulates during intracellular development until merozoite release. We show that GAP45 is phosphorylated by calcium dependent protein kinase 1 (CDPK1), and identify the modified serine residues. Replacing these serine residues with alanine or aspartate has no apparent effect on GAP45 assembly into the motor protein complex or its subcellular location in the parasite. The early assembly of the motor complex suggests that it has functions in addition to its role in erythrocyte invasion.

## Introduction

Malaria is a disease caused by protozoan parasites of the genus *Plasmodium* and results in almost a million deaths annually [1]. The life cycle is complex with alternate stages in a vertebrate host and a mosquito vector. In the asexual cycle in the host's blood stream the merozoite form of the parasite invades a red blood cell and develops into the so-called trophozoite. During subsequent schizogony, DNA replication and mitosis results in a multinucleate syncytium, this then undergoes cytokinesis or segmentation to produce new merozoites that are released to invade red blood cells. Segmentation is accompanied by the formation of the inner membrane complex (IMC), a series of flattened cisternae that are found immediately beneath the parasite plasma membrane (PM) [2]. The IMC may provide shape, rigidity and polarity to the developing merozoites, which bud off from the residual body prior to their release from the red cell. Polarity is also established by the synthesis and location of a set of apical organelles that participate in merozoite release and host cell reinvasion. Host cell invasion is an active process powered by an actin-myosin motor complex located between the parasite's PM and the IMC. Myosin is tethered to the IMC and during invasion moves filamentous (F) actin to the rear of the parasite. The actin filament is coupled to a junction involving the parasite PM and the host cell surface membrane via transmembrane adhesins, thus the action of the molecular motor results in forward motion of the parasite into the host cell (reviewed in [3], [4]).

The motor complex consists of myosin A (MyoA, a type XIV myosin), a myosin light chain (called myosin tail domain-interacting protein (MTIP) in *Plasmodium*) and the glideosome associated proteins GAP50 and GAP45, which were first described in *Toxoplasma gondii* [5]. GAP50 has a signal peptide and is targeted to the IMC through the protein secretory pathway, whereas GAP45, MTIP and MyoA are translated on cytoplasmic ribosomes and form a complex cotranslationally [5], [6]. The GAP45-MTIP-MyoA complex subsequently binds to GAP50, presumably at the IMC. Whilst GAP50 has been used as a marker for the development of the IMC [7], the assembly of the other elements of the myosin motor complex and the timing of their association with the IMC is unclear.

In *Plasmodium falciparum*, the most important human malaria parasite, GAP45 is expressed throughout schizogony and, in complex with MyoA, MTIP, and GAP50, accumulates and is abundant in late schizont stages [8]. Although PfGAP45 is only 204 amino acids, it migrates anomalously as multiple bands on SDS-PAGE, suggestive of post-translational modification. The protein is cotranslationally N-myristoylated and then palmitoylated [6]. In addition, GAP45 is phosphorylated *in vivo* and during the progression of schizogony the proportion of GAP45 that is phosphorylated increases [9]. It has been reported to be a substrate for calcium-dependent protein kinase 1 (CDPK1) and protein kinase B (PKB) *in vitro* thus highlighting the potential importance of multiple kinases in regulating either the formation or the function of the parasite motor complex [9], [10]. Both CDPK1 and PKB are regulated by calcium, consistent with an important role for calcium flux in regulating *Plasmodium* growth and invasion [11].

Two phosphopeptides have been isolated from GAP45 purified from *P. falciparum* merozoites (residues 81–96 and 141–155). These contain threonine and/or serine residues that may be phosphorylated by serine/threonine-specific protein kinase(s). In addition to peptide 81–96, CDPK1 also phosphorylated GAP45 *in vitro* on a single residue contained within the peptide 97–112 [9]. These regions of GAP45 are conserved across the *Plasmodium* genus, but this conservation does not extend to the GAP45 sequence in other Apicomplexan parasites such as *T. gondii*. The phosphorylation of GAP45 in *P. falciparum* starts from approximately 36 hours post invasion and increases throughout schizogony [9]. Furthermore, pulse chase studies suggest that GAP45 is phosphorylated before GAP50 joins the complex [6]. In *T. gondii* tachyzoites, phosphorylation of GAP45 at S163 and/or S167 has been shown to regulate association/dissociation of the motor complex [12], however these residues are in a poorly conserved region and are not present in PfGAP45. These issues need to be clarified, however current evidence suggests that post-translational modifications such as phosphorylation may be important for localisation of, and interaction between, proteins of the motor complex.

The mechanism and timing of motor complex formation and localisation is not clearly defined. During schizogony, nuclear division is accompanied by IMC development [13]. In early schizonts (with up to eight nuclei) integral membrane protein markers of the IMC such as GAP50, GAPM1 and GAPM2 are located at what are variously described as punctate, ring-like, doughnut-shaped, or clamp-like structures at the periphery of the parasite [7], [14], [15]. As the schizont develops further, these IMC-specific proteins are detected extending around the surface of the developing merozoites. In *T. gondii*, recruitment of motor complex proteins to the pellicle may also occur prior to segmentation to produce daughter cells [16], [17]. However in *T. gondii*, cell division occurs via endodyogeny, a process distinct to schizogony [13], and during which distribution of motor complex proteins is not identical between mother and daughter tachyzoites. TgGAP50 and TgGAP40 (a recently identified IMC-associated protein) are found in the IMC of both mature parasites and immature daughter cells whereas TgMyoA, TgMLC1, and TgGAP45 are present at the IMC of mature parasites yet are entirely absent from developing daughter cells [5], [17].

We wished to determine whether or not GAP45 is associated with the developing IMC throughout schizogony in *P. falciparum*, if sequences at the N-terminus are sufficient to target GAP45 to the IMC, and whether phosphorylation of the serine residues that are substrates for CDPK1 is important in the assembly and location of the motor complex. We have demonstrated that a GFP-tagged full length GAP45 can be expressed in the parasite and is targeted to the same location as the untagged form. We identified the GAP45 residues phosphorylated by CDPK1 *in vitro* and were able to examine the effect on both subcellular location and formation of the motor complex of substituting these residues with either alanine or aspartic acid in parasite-expressed GFP-tagged GAP45.

## Results

### The role of the N-terminus of PfGAP45 in protein localisation

The two acylation sites at the N-terminus of PfGAP45, G2, which is myristoylated, and C5, which is presumed to be palmitoylated, have been suggested to drive the localisation of GAP45 to the IMC [6]. However, calcium-dependent protein kinase-1 (CDPK1), another protein with a similar dual acylation motif, has been demonstrated to be targeted to the inner face of the plasma membrane of *P. falciparum* merozoites [9]. We wished to establish if the N-terminal 29 residues of GAP45 were sufficient to target a reporter protein, GFP, to the IMC. We compared the localisation of this extremely truncated GAP45 (N-GAP45) with that of full-length GAP45 in which GFP was inserted into the ORF just after the N-terminal 29 amino acids (FL-GAP45), and also with a construct that lacked the N-terminus of GAP45 and therefore the putative membrane targeting residues at the N-terminus (C-GAP45). We chose to introduce the GFP tag at an internal site of GAP45 as previous work in *T. gondii* revealed that the addition of a YFP tag to the C-terminus of TgGAP45 abrogated its binding to the rest of the motor complex [18] and only an internally located epitope tag was able to circumvent this problem [12].

**Figure 1:**
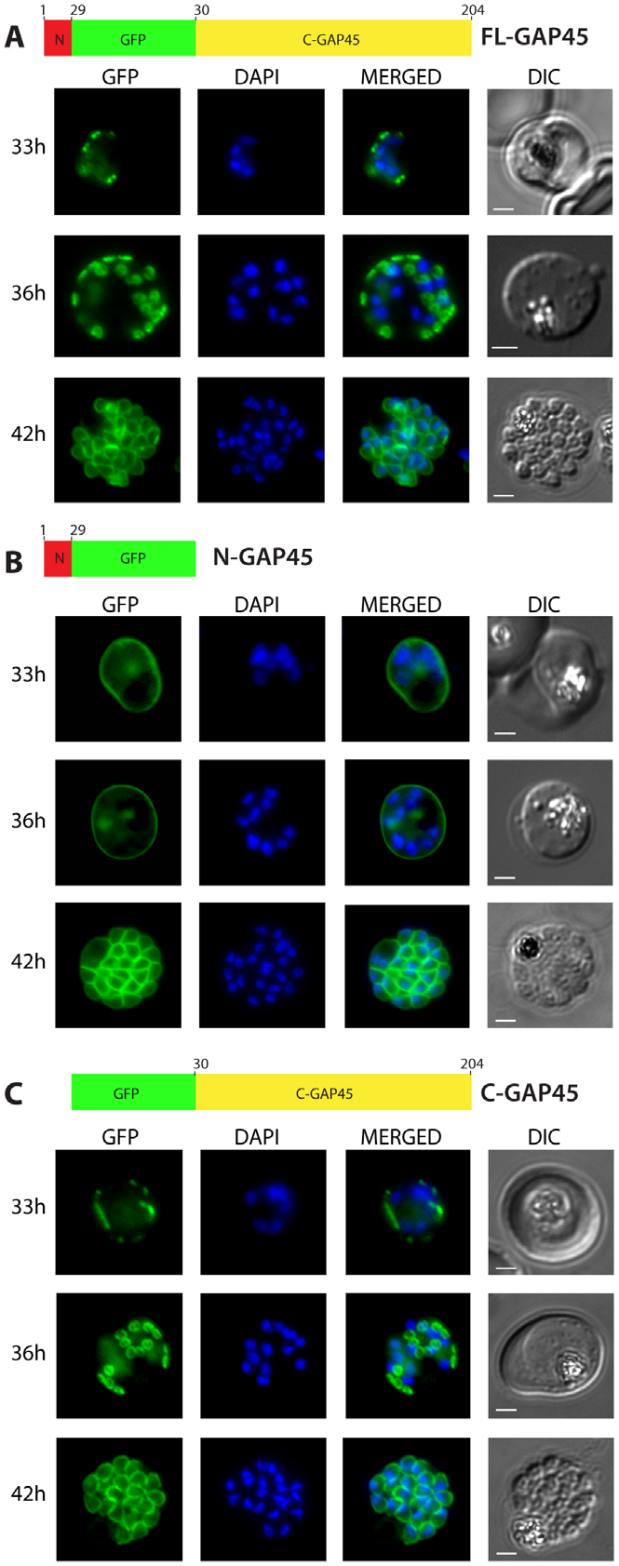
Subcellular localisation of GFP-tagged GAP45 protein and truncated derivatives. The location of (A) GFP-tagged wild type GAP45 (FL-GAP45), (B) GFP-tagged C- terminally truncated GAP45 (N-GAP45), and (C) GFP-tagged N-terminally truncated proteins (C-GAP45) during schizont development. Transfected parasite populations were synchronized and examined at 33, 36 and 42 h post invasion by fluorescence microscopy to detect GFP (green). Nuclei were labelled with Hoechst stain (blue) prior to analysis. The merged image of the two is also shown together with the corresponding differential interference contrast (DIC) picture. Scale bar is 2 µm. In the early schizont stages small, localised structures of GFP signal were observed around the parasite's periphery in both FL-GAP45 and C-GAP45, typical of an IMC location. N-GAP45 protein produced a different pattern of fluorescence probably corresponding to the parasite plasma membrane. At the late schizont stage (42 h post invasion) the GFP signal was detected at the periphery of merozoites developing within the schizont for all GAP45 variants, and at this time point the putative IMC and parasite plasma membrane patterns were indistinguishable.

Live fluorescent imaging of these parasites revealed striking differences between the localisation of N-GAP45 and the other proteins. FL-GAP45 localises to ring-like structures in the developing schizonts (Figure 1A, 33 and 36 h) that appear to be present towards the periphery of the parasite. In fully segmented schizonts, the protein fully surrounds each merozoite nucleus (Figure 1A, 42 h). By contrast, in early schizonts the fluorescence of N-GAP45 was more evenly distributed around the periphery of the entire parasite rather than in discrete foci and there were no ring-like structures evident (Figure 1B, 33 and 36 h). In mature, segmented schizonts N-GAP45 also was located around the periphery of each merozoite (Figure 1B, 42 h). Interestingly, the localisation of C-GAP45, lacking the N-terminal acylation motifs, resembled very closely that seen for the FL-GAP45: in early schizonts the protein was present in discrete foci (Figure 1C, 33 h) that progress to small ring-like structures as development continues (Figure 1C, 36 h) and finally in the segmented schizont the protein was evenly distributed around the periphery of each merozoite (Figure 1C, 42 h). We confirmed by immunofluorescence using antibodies raised against GAP45 that the endogenous protein is also located to ring-like structures during schizogony in the 3D7 parental parasite line (time course from 30 h to 42 h post-invasion shown in Figure S1A) and that FL-GAP45 and C-GAP45 fusion proteins colocalise exactly with endogenous GAP45 in immature and mature parasites, whereas N-GAP45 does not (Figure S1B).

**Figure 2:**
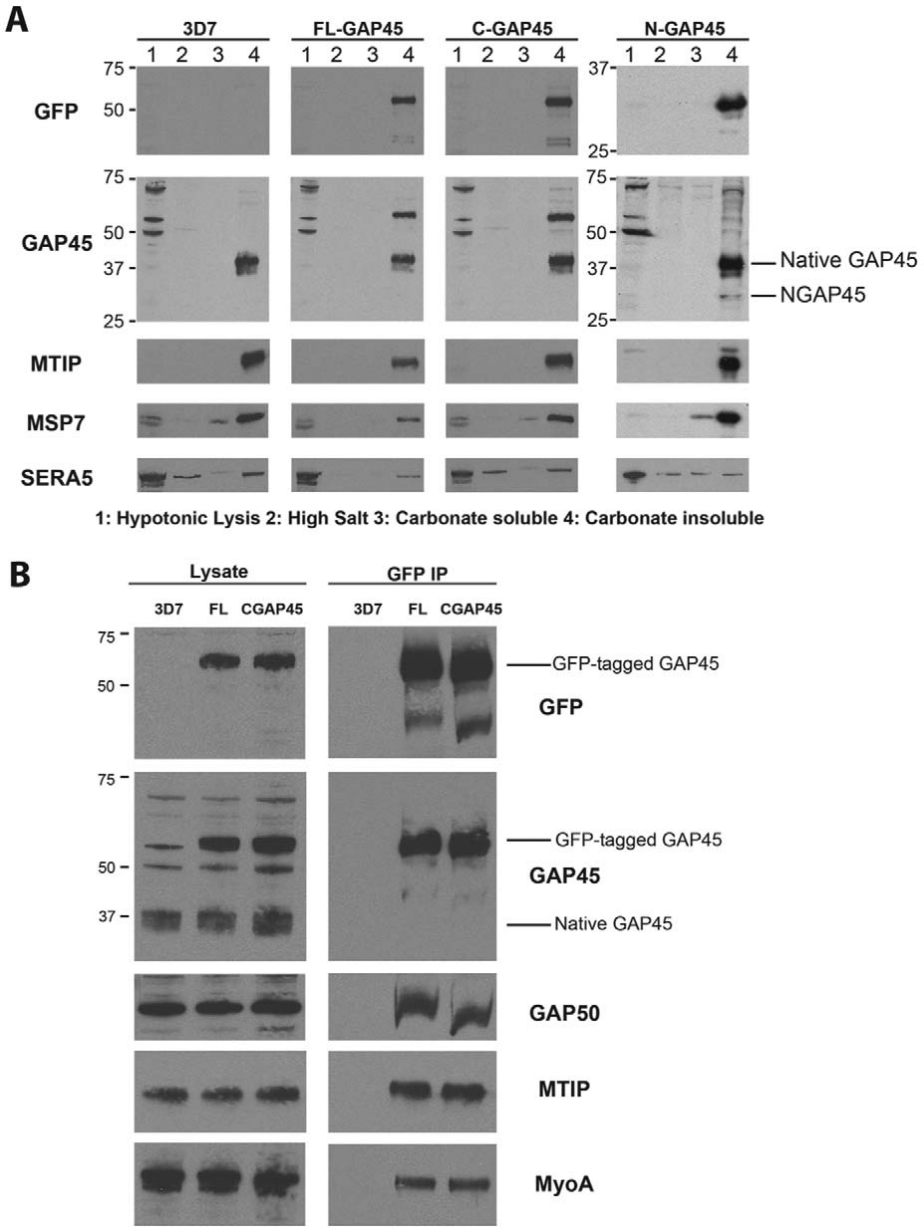
Endogenous GAP45 and GFP-tagged variants are membrane-associated proteins. (A) The subcellular fractionation of untransfected parasites (3D7) and parasites expressing FL-GAP45, C-GAP45 or N-GAP45 was performed sequentially, by (1) hypotonic buffer lysis, (2) high salt buffer, and (3) sodium carbonate buffer; the residual material (4) is the carbonate-insoluble pellet. All fractions were analysed by SDS-PAGE and Western blotting, using antibodies to GFP, GAP45, MTIP, MSP7, and SERA5. GAP45 proteins were all found in the carbonate-insoluble pellet. (B) Immunoprecipitation of GFP-fusion proteins followed by Western blotting demonstrates that both FL-GAP45 and C-GAP45 form a complex containing MyoA, MTIP and GAP50.

To determine if all three GFP-fusion proteins were targeted to membranes of the parasite cellular proteins were extracted in a series of buffers designed to solubilise proteins based on their degree of membrane association [19]. Only integral membrane proteins or those extremely tightly associated with membranes would fail to be solubilised by a high pH carbonate buffer. All three GAP45-GFP proteins were insoluble in high pH carbonate buffer (Figure 2A), as was MTIP, another member of the motor complex. MSP7, a peripheral membrane protein was slightly solubilised by carbonate buffer with most of the protein remaining insoluble. SERA5, a protein of the parasitophorous vacuole that is not membrane associated is largely released upon parasite treatment with a hypotonic solution (lane 1). Thus our data suggest that the N-terminus of GAP45 is sufficient to target a protein to cell membranes, however a protein lacking this sequence (C-GAP45) is still tightly associated with the membrane fraction. We wished to determine whether or not this truncated GAP45 protein was able to form part of the motor complex with MyoA, MTIP and GAP50. Immunoprecipitations of GFP fusion proteins were performed followed by Western blotting with antibodies to components of the motor complex. Both FL-GAP45 and C-GAP45 coprecipitated MyoA, MTIP and GAP50 but not native GAP45 (Figure 2B). These results suggest that the C-terminus of GAP45 is responsible for binding to other protein components of the motor complex. The absence of native GAP45 in the precipitates also suggests that the GAP45 proteins do not form homo-oligomers and that there are not multiple copies of GAP45 within a single motor complex.

### The N-terminus of GAP45 targets proteins to the plasma membrane but is not required for the association of GAP45 with the IMC

**Figure 3:**
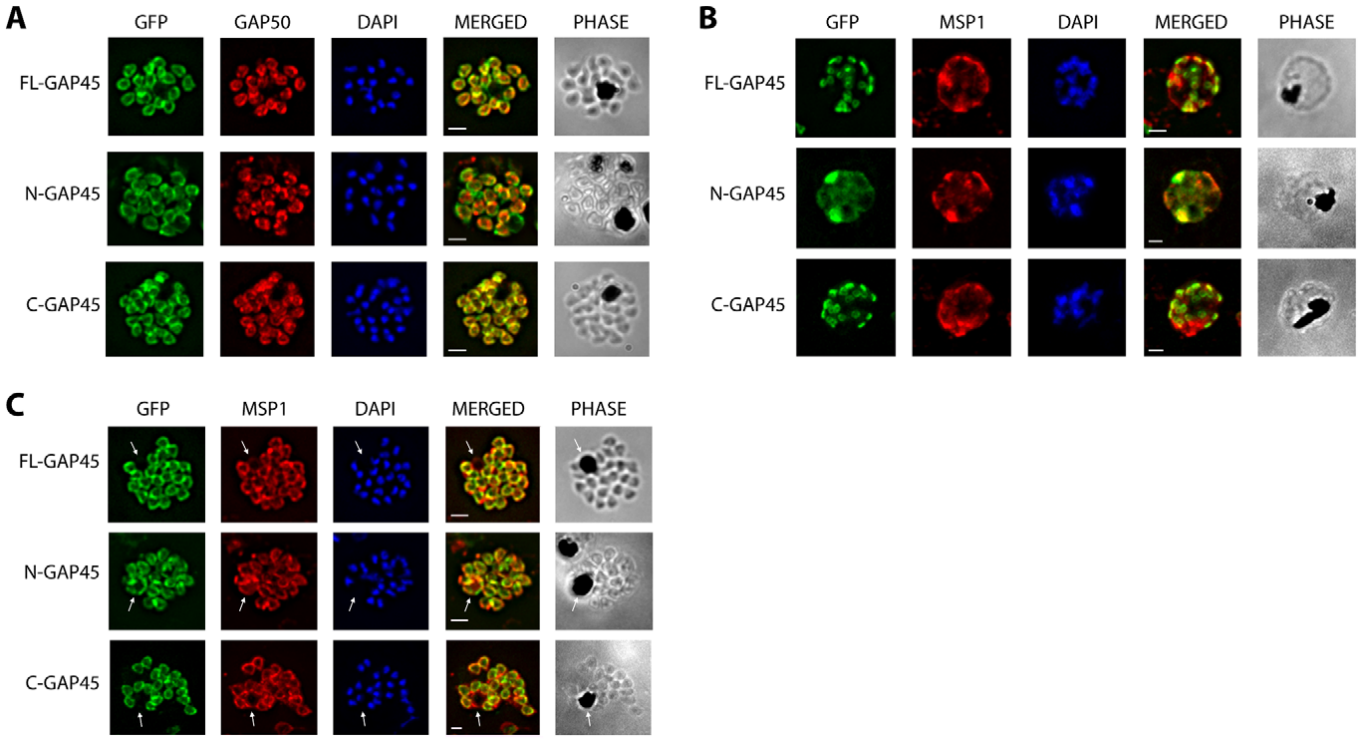
Immunofluorescent staining of GFP-tagged GAP45 variants with GAP50 in late stage schizonts. (A) Parasites were fixed and the location of GFP (green) and GAP50 (red), examined. FL-GAP45, C-GAP45 and N-GAP45 all appear to colocalise with GAP50 in segmented schizonts. (B) Staining with anti-MSP1 antibody (red) colocalises with only N-GFP in developing schizonts. FL-GAP45 AND C-GAP45 are present in distinctive ring-shaped structures at the periphery of the developing schizont. (C) In mature schizonts MSP1 (red) is present on membranes surrounding the residual body. N-GAP45 is also present on the residual body, but FL-GAP45 and C-GAP45 are absent. The white arrow in all images indicates the residual body. These data are consistent with a plasma membrane location for N-GAP45 and an inner membrane complex location for FL-GAP45. In all cases, parasite nuclei were stained with DAPI (blue); merged images are also shown. Scale bar is 2 µm.

Whilst the N-GAP45 construct was shown to be membrane associated (Figure 2A), it is clear from cell imaging that it is targeted to a different membrane than FL-GAP45 and C-GAP45 proteins (Figure 1). We sought to clarify which peripheral membrane each protein is targeted to by immunofluorescence using antibodies to proteins of known membrane localisation. Merozoite surface protein 1 (MSP1) is a GPI-anchored protein of the parasite plasma membrane (PM); whereas glideosome associated protein 50 (GAP50) is an integral membrane protein of the IMC. All three of our GFP-fusion proteins localise to the periphery of mature, fully segmented schizonts and seem to colocalise with the IMC-specific marker GAP50 (Figure 3A), however such staining is indistinguishable from that of PM-specific proteins. In younger, unsegmented schizonts the picture is different; N-GAP45 colocalises with MSP1 at the PM, whereas the staining pattern of FL-GAP45 is distinct (Figure 3B).

In mature schizonts it is difficult to distinguish between the PM and IMC as they are so close together. Late in schizogony individual merozoites are pinched off from the syncytium and the residual body is encapsulated by plasma membrane, but not IMC. Residual bodies therefore stain for plasma membrane proteins (e.g. MSP1), but not IMC proteins (e.g. GAP50). Close examination of the residual bodies of mature schizonts expressing FL-GAP45, C-GAP45 or N-GAP45 revealed that only N-GAP45 was present around residual bodies (marked by arrows on Figure 3C), indicating a plasma membrane location for the truncated GAP45 and an IMC localisation for FL-GAP45 and C-GAP45.

### CDPK1 phosphorylates S89 and S103 of PfGAP45

**Figure 4:**
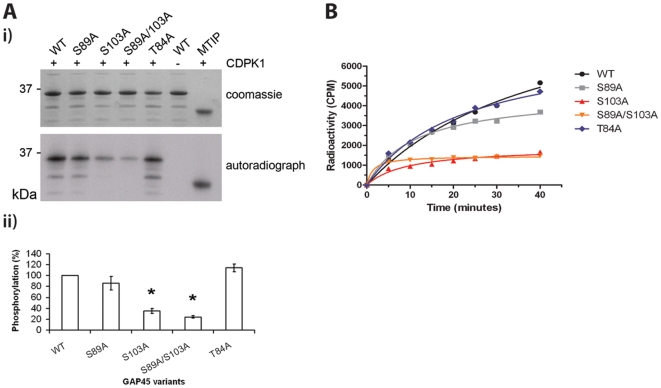
Identification of CDPK1 phosphorylation sites in GAP45. (A i) *In vitro* CDPK1-mediated phosphorylation of recombinant PfGAP45 (WT) and the S89A, S103A, and the T84A variants using 100 nM CDPK1 and ɣ^32^P-ATP at 30℃, for 10 min. MTIP was included as a substrate control for CDPK1. The upper panel shows the coomassie blue stained gel, and the lower panel shows the autoradiograph of the same gel. (A ii) The intensity of the phosphorylated GAP45 signal (autoradiography) was standardized against the amount of GAP45 protein (Coomassie blue stained) and analysed by ImageJ software. The data are presented as a mean ± S.E.M. from 3 to 5 different experiments. *denotes a significant difference (p<0.05) compared to wild type. (B) A time-course of CDPK1-dependent phosphorylation of recombinant GAP45 proteins by measuring incorporation of ^32^P at 30℃, using 100 nM CDPK1 and 0.1 mM ɣ^32^P-ATP and detection by scintillation counting. The data are presented as a mean from duplicate reactions for the unmodified protein (WT) or variants where S89 and/or S103 are replaced by alanine. T84A is included as a control as it is a substitution that had no effect on phosphorylation by CDPK1.

We sought to further investigate the targeting and complex formation of GAP45 by identifying residues of the protein that are phosphorylated, specifically by CDPK1. Phosphopeptides derived from GAP45 have been identified from late schizonts [20] and merozoites [9], and phosphorylation of residues 163 and/or 167 of *T. gondii* GAP45 has been shown to be crucial for binding of the trimeric complex of GAP45, MTIP and MyoA to the IMC protein GAP50 [12]. We undertook a rigorous site-directed mutagenesis approach to identify specific residues of GAP45 that were phosphorylated by CDPK1 *in vitro*. As there is no detectable threonine phosphorylation of GAP45 by CDPK1 (based on phosphothreonine-specific antibody binding, data not shown), we restricted our analysis to the sixteen serine residues of GAP45. Each serine was mutated to an alanine, and the ability of CDPK1 to phosphorylate wild type and variant GAP45 proteins was examined using γ-^32^P-labelled ATP incorporation. By autoradiography, all of the GAP45 variants except S103A had a similar intensity of labelling as the wild type GAP45 protein (Figure 4A and Table S1). Less incorporation of label was observed for the S103A protein, and by densitometry analysis of data from at least three different experiments the phosphorylation of the S103A variant was about 35% of that for the wild type protein, representing a significant decrease (p<0.05). In addition, the S31A, S89A and S156A variants also showed slight decreases in phosphorylation of 10, 14 and 13% respectively when compared to the wild type protein, although these values did not represent statistically significant differences (Fig. 4A and Table S1).

CDPK1 phosphorylation of wild-type GAP45 and the S89A, S103A and S89A/S103A variants was also examined as a time course with samples collected at sequential time points and analysed by scintillation counting (Figure 4B). The results from this assay confirmed that S89 and S103 are indeed targets of CDPK1 activity *in vitro*, with decreased ^32^P incorporation, although at early time points the incorporation of ^32^P into S89A GAP45 was indistinguishable from that into wild type GAP45. These findings complement the autoradiography results and are consistent with the *in vivo* data identifying phosphopeptides of GAP45 containing these residues [9], [20]. Phosphorylation of the S89A/S103A variant was also decreased compared to that of wild type GAP45, but the double substitution showed only 5% less ^32^P incorporation than the S103A variant (Figure 4B). In the case of this double mutant GAP45, there was still 24% phosphorylation compared to the wild-type protein. The phosphorylated residues are likely to be S31 and S156, identified as possible CDPK1 targets in the analysis of individual serine substitutions of GAP45 (Table S1). Since this work has been performed, an analysis of the *P. falciparum* phosphoproteome revealed multiple phosphorylated residues of GAP45, including S89, S103 and also S156, identified here as a minor CDPK1 site [20]. Our *in vitro* kinase assays verify that three of the GAP45 phosphosites identified by Treeck *et al* in their phosphoproteome analysis are substrates for CDPK1. Whilst this does not confirm that these phosphorylations are mediated by CDPK1 *in vivo*, the fact that the two proteins colocalise at the inner face of the plasma membrane strengthens the argument that this may also happen in the parasite. Our *in vitro* studies have also demonstrated that five of the eight sites identified by Treeck are not phosphorylated by CDPK1 *in vitro*, thereby implicating other kinases in the modification of this protein. We elected to focus further efforts on analysis of the role of S89 and S103 phosphorylation by CDPK1 *in vivo*, as S103 is the major CDPK1 site in GAP45 and S89 phosphopeptide had previously been isolated from merozoites [9].

### Phosphorylation of S89 and/or S103 has no effect on trafficking or assembly of the motor complex

Having shown that GFP-tagged GAP45 behaves like native GAP45 in terms of its location in the cell throughout schizogony, we were next interested to examine the effect of modifying S89 and S103 in GFP-tagged GAP45 on its behaviour in the cell. Therefore we constructed plasmids for the episomal expression of GFP-tagged GAP45, in which either or both S89 and S103 were modified to Ala or to Asp. Ala was chosen to obviate a negative charge at the sequence position and Asp was chosen to introduce a phosphomimetic, permanently negatively charged residue at the position.

**Figure 5:**
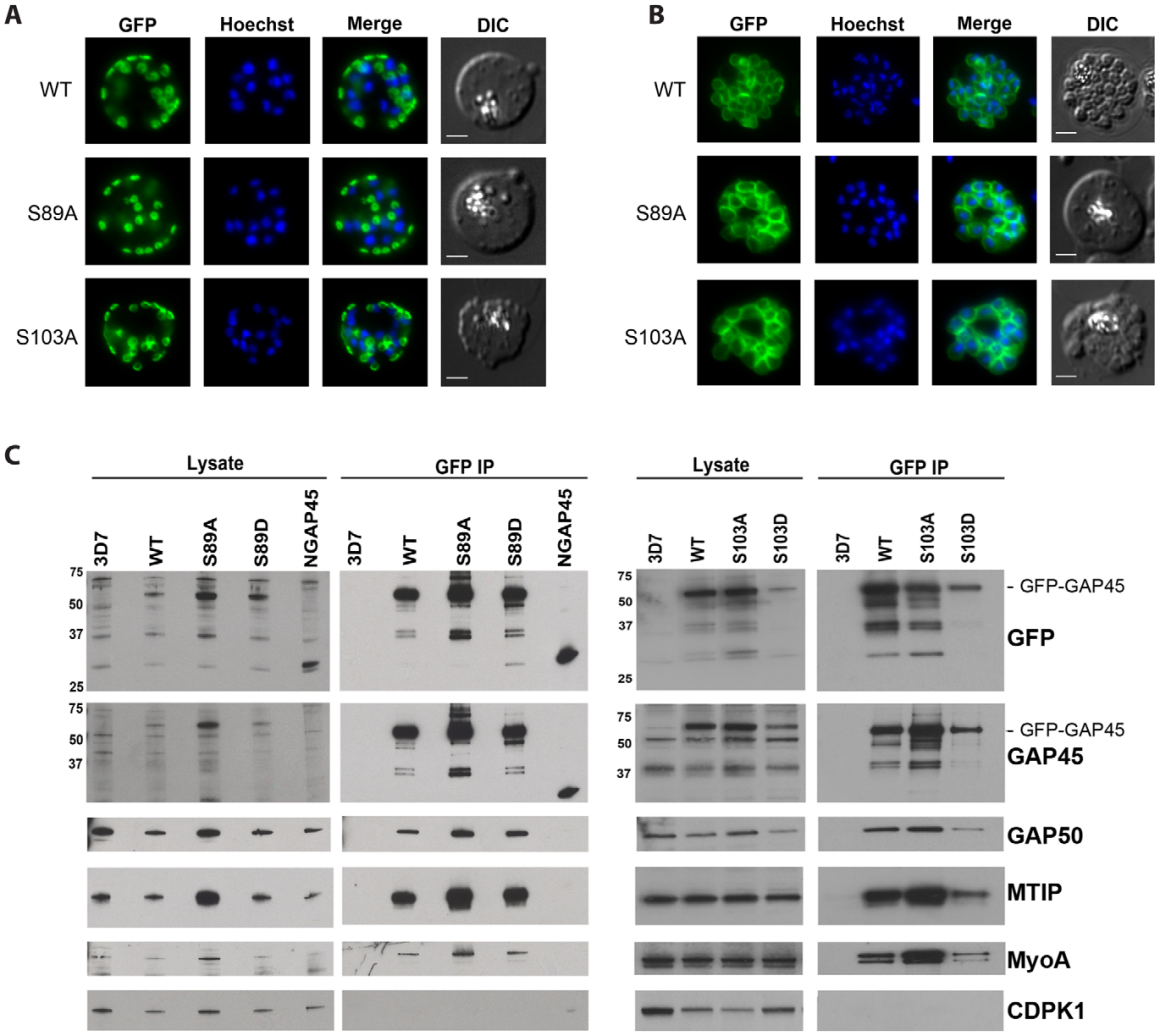
Mutation of S89 and/or S103 has no effect on subcellular localisation of GAP45 or its inclusion in the motor complex. The location of WT GFP-tagged GAP45, and mutants S89A and S103A determined in (A) early and (B) late schizonts, by live fluorescence microscopy (green). Parasite DNA was stained with Hoechst dye (blue); merged images and the differential interference contrast pictures are also shown. Scale bar is 2 µm. In the early schizont stages small, localised regions of GAP45GFP signal were observed around the parasite's periphery, typical of an IMC location. At the late schizont stage the GFP signal was detected at the periphery of merozoites developing within the schizont. There was no difference in the pattern of location between the wild type (WT) protein and any of the mutants (see also Figure S2). (C) Detergent lysates of schizont stage-parasites were prepared from untransfected 3D7 or parasites transfected with plasmid to express the WT, S89A, S89D, S103A and S103D mutant proteins. N-GAP45 (comprising the first 29 amino acids of GAP45 fused to GFP) was included as a negative control. Complexes were immunoprecipitated with an anti-GFP monoclonal antibody. Precipitated proteins and a sample of the corresponding whole-cell lysate were resolved by SDS-PAGE and analysed by western blotting using antibodies to GFP, GAP45, GAP50, MTIP, MyoA and CDPK1. CDPK1 is known to not be part of the motor complex and was therefore used as a negative control. The mobility of standard proteins is indicated on the left side of the gels. The results show that, with the exception of N-GAP45, all of the GFP-tagged GAP45 proteins are in complex with MTIP, MyoA and GAP50.

By live microscopy, the S89A and S103A modified GAP45 proteins were indistinguishable in their location from unmodified GAP45 in both early (Figure 5A) and late schizont (Figure 5B) stages. Neither the single Ala or Asp variants nor the double S89A/S103A and S89D/S103D substitutions had any effect on GAP45 localisation (Figure S2). As before, we immunoprecipitated GFP-tagged GAP45 from schizont lysates. By Western blotting each of the mutant proteins was found in complex with MyoA, MTIP and GAP50 and, as such, were indistinguishable from parasites expressing wild type GAP45 (S89A and S103A are shown in Figure 5C).

## Discussion

The subcellular distribution that we see of a GFP-tagged GAP45 protein appears to be identical to the pattern of GAP50 expression observed in developing schizonts, where claw-shaped structures were seen [7], and that of other membrane localised IMC proteins GAPM1 [15] and GAPM2 [14]. These observations fit with a recent model of the motor complex in *T. gondii* tachyzoites where GAP45 is anchored in the plasma membrane by dual acylation of its N-terminus and interacts with GAP50 in the IMC membrane via its C-terminus. Whether this interaction is direct, or via other members of the motor complex (MTIP or MyoA, or the recently described GAP40) has not been determined. We have demonstrated that whilst the N-terminus of GAP45 containing the dual acylation motifs is able to target a reporter protein to the plasma membrane, it is not required for localisation and trafficking of the motor complex to the IMC; a protein lacking the N-terminus shares the same IMC localisation as FL-GAP45 and is able to form a tetrameric complex with MyoA, MTIP and GAP50. It is likely that we see no growth or structural phenotype as a result of this because the GAP45-GFP proteins in our studies are expressed from episomal plasmids and there is always native GAP45 expressed from the genomic locus in our parasites. However, we can say that there is no obvious detrimental effect on parasites in which a considerable proportion of the motor complex is not anchored to the plasma membrane via GAP45.

During schizogony, cell replication is initiated with nuclear division and replication of other organelles developing within the cytoplasm, encapsulated by a single plasma membrane from the mother cell [13], [15]. After organelle replication merozoites are pinched off from the syncytium within the red blood cell by the plasma membrane surrounding individual merozoites. Our data suggest that even at very early stages of merozoite formation, before plasma membrane formation around individual merozoites, GAP45 interacts with proteins in the developing IMC membranes. If the motor complex is only required for merozoite invasion of red blood cells why is it assembled much earlier in the developing schizont? It appears that IMC formation occurs concurrently with plasma membrane encapsulation of daughter merozoites. It is likely that a molecular motor is involved in this process. In other systems, myosins are crucial for cell division, for example myosin II is the primary motor protein responsible for cytokinesis in eukaryotes [21]–[23]. Notably, in another Apicomplexan parasite, *T. gondii* myosin B has been implicated in the process of cell division [24]. Because of the unusual arrangement of membranes in the development of merozoites, it may be that *Plasmodium* achieves cytokinesis and segmentation using a myosin XIV in a motor complex that effectively brings the plasma membrane and IMC together by virtue of a member of the complex, GAP45, bridging the gap between the two membranes.

As the expression of both CDPK1 and GAP45 peaks late in schizogony [9], it is possible that phosphorylation of GAP45 is important in the regulation of motor complex formation. We have shown that mutation of either or both S89 and S103 to alanine or aspartate does not affect the localisation of GAP45 to the IMC throughout all stages of schizogony. Nor do these mutations affect the ability of GAP45 to form the tetrameric complexes with MyoA, MTIP and GAP50. Phosphorylation of sites other than S89 and S103 may be important in actin-myosin motor complex assembly. The localisation of GAP45 to the IMC has also been shown to be unaffected by phosphorylation in another Apicomplexan parasite, *T. gondii*. However, phosphorylation of *T. gondii* GAP45 does causes dissociation of the trimeric MyoA-MTIP-GAP45 complex from GAP50 [12]. The phosphorylated residues in TgGAP45 (S163 and S167) are in the least conserved region of the protein and are not identical in *P. falciparum* GAP45; the equivalent positions are D117 and T121. The TgGAP45 mutation S163E causes dissociation of the motor complex [12], whereas the equivalent position in PfGAP45 is an acidic aspartate, suggesting that mechanisms of regulation of motor complex formation may not be conserved between the two species, or that negative charge alone was not the cause of complex dissociation in the TgGAP45 mutant protein. GAP45 has been shown to be multiply phosphorylated in vivo, with residues 107, 142, 149, 156, 158 and 198 being modified as well as serines 89 and 103 [20]. It may be that phosphorylation of one or more of these residues play an equivalent role to that of S163 *T. gondii*. Phosphorylation of PfGAP45 on S89 and S103 may be crucial during the invasive asexual stage of *P. falciparum*, the merozoite, or just after invasion in very early ring stages. At this stage, during or just after red blood cell invasion, the disassembly of the motor complex may be more critical, and phosphorylation/dephosphorylation of component proteins may play a role in this process. Alternatively, phosphorylation of these residues may have a completely different consequence that was not evident in our studies.

In conclusion, the present investigation has shown that phosphorylation of S89 and S103 residues on GAP45 does not affect the IMC localisation or the formation of the tetrameric motor complex during schizogony. Our studies have shed light on the processes of merozoite formation within the multinucleate syncytium and suggest that motor complex formation at the IMC occurs in parallel with cytokinesis and segmentation, a process that warrants closer scrutiny.

## Materials and Methods

### Ethics statement

The National Blood Transfusion Service provided human O+ erythrocytes from anonymous volunteers. Transfusion bags are bar coded and no records have been received linking individual donors to blood donations.

### Parasite growth, synchronisation and transfection

*P. falciparum* parasites were maintained in human O+ erythrocytes provided by the National Blood Transfusion service. 3D7 is a cloned line derived from NF54 obtained from David Walliker at Edinburgh University [25]. Parasites were cultured in RPMI 1640 medium supplemented with 1% Albumax at 3% hematocrit in gassed (90% nitrogen, 5% oxygen, 5% carbon dioxide) flasks at 37°C. Parasites were synchronized using Percoll gradient centrifugation as previously described [26]. For transfection, *P. falciparum* 3D7 parasites were synchronized as described [26] and used at 10% ring stage parasitemia. Transfection vector DNA (100 µg in 100 mM Tris-HCl, 10 mM EDTA, pH 8.0 [TE] buffer) was transfected into parasites at ring stage as described [27], [28]. Parasites were then grown in a medium containing 2.5 µg/ml blasticidin (Merck).

### Live and indirect-immunofluorescence microscopy assay

Live synchronized parasite populations expressing GFP-tagged proteins were examined by epifluorescence microscopy on an Axio Imager M1 microscope (Zeiss). Parasites were labelled with Hoechst 33342 (Invitrogen) DNA stain, at 1 µg/ml, then a cell suspension was placed on a slide and overlaid with a Vaseline-rimmed coverslip. For immunofluorescence assays (IFA), thin smears of parasites were fixed with 4% paraformaldehyde for 10 min at RT. The fixed cells were washed 3 times with PBS, permeabilised with PBS containing 0.1% Triton X-100 for 5 min followed by blocking in 4% BSA (w/v) in PBS at 4°C overnight. After blocking, the slides were incubated for 1 h at RT with GFP-booster (a specific GFP-binding protein coupled to the fluorescent dye ATTO 488; Chromotek) followed by washing with PBS and addition of an antigen specific primary antibody. The slides were then incubated with IgG-specific secondary antibody coupled with Alexafluor 594 (Sigma) for 1 h at RT and washed 3 times with PBS. The smears were mounted for microscopic examination with Prolong® Gold antifade reagent with DAPI (Invitrogen). Images were acquired on a DeltaVision Core system (Applied Precision Inc., USA) based on an Olympus IX71 inverted microscope, using an Olympus 100× objective lens and images captured using a QuantEM 512SC EMCCD (Cascade 512×512) camera (Photometrics Ltd) using a Xenon light source. Images were deconvoluted using DeltaVision SoftWorx software suite 5.0 and prepared for publication with Adobe Photoshop.

### Plasmid construction

The plasmid phh3-bsd-NGAP45GFP was designed to episomally express the N-terminal 29 residues of GAP45 fused to GFP. DNA sequence encoding the N-terminal 29 amino acids of GAP45 was fused in front of sequence encoding GFP as follows: Two overlapping primer pairs (Gap45for[TTCACCTAGGATGGGAAATAAGTGTAGTAGGTCAAAGGTAAAAGAACCAAAGAGAAAAGATATTGACGAGTTAGCAGAAAGGGAG]/Gap45ev[GACAACTCCAGTGAAAAGTTCTTCTCCTTTACTTTTCTTCAAGTTCTCCCTTTCTGCTAACTCGTCAATATC]) were fused to generate a long forward primer encoding the N-terminal 29 amino acids of GAP45. Following this, the long primer was used in combination with GFPrev primer (ATTTCTCCGCGGTTATTTGTATAGTTCATCCATGAAATGTGTAATCCC) to produce the GAP45-GFP fusion gene, which was subsequently inserted between the MSP3 promoter and the *hrp2* 3′UTR sequences in the pHH3 vector (Knuepfer and Holder, unpublished).

A second plasmid (phh3-bsd-GFPGAP45) was constructed to add an internal GFP tag between residues 29 and 30 of the GAP45 sequence. The sequence encoding the N-terminal part of GAP45 fused with GFP was amplified by PCR, eliminating the terminal stop codon using primers 5′ GCGCGCCCTAGGATGGGAAATAAATGTTCAAG 3′ and 5′ GCGCGCCCGCGGTTTGTATAGTTCATCCATGC 3′. The PCR product was reinserted into the pHH3 vector after the MSP3 promoter. The sequence encoding the C-terminal portion of GAP45 from residue 30 to the stop codon was amplified by PCR using primers 5′ GCGCGCCCGCGGCAATCTGAAGAAATAATTGAAG 3′ and 5′GCGCGCCCGCGGTTAGCTCAATAAAGGTGTATCG 3′ and inserted into the vector after the GFP tag sequence. A series of additional constructs were also made to express the full length GAP45 tagged with GFP and with amino acid substitutions (see below).

### Production of GAP45 and variant proteins

The GAP45 open reading frame was cloned into the pET46 Ek/LIC expression vector (Novagen), containing an N-terminal hexa-His tag and transformed into BL21 (DE3) RIL competent cells (Stratagene). Protein expression was induced by the addition of 1 mM IPTG for 3 h at 27°C, then pelleted cells lysed and extracted in Bugbuster solution (Novagen) containing 1× protease inhibitor cocktail without EDTA (Roche) and benzonase (25 U/ml) (Novagen). The His-tagged GAP45 protein was then purified from lysates on Nickel-Nitrilotriacetic acid (Ni-NTA) agarose (Qiagen).

To produce variant proteins the gene sequence was first modified using a QuikChange II Site-Directed Mutagenesis kit (Stratagene) according to the manufacturer's instructions. All serine residues were replaced individually with Ala and additional variants with up to three replacements to Ala were also made. Variants S89A, S103A, S89A/S103A, S89D, S103D, and S89D/S103D were also prepared for expression in the parasites as GAP45GFP protein. HPLC-purified complementary oligonucleotides containing the required changes were purchased (Sigma) and are described in Table S1. Mutagenesis reactions were performed with 10 ng plasmid DNA, according to the manufacturer's instructions. The presence of the desired mutation was confirmed by sequencing of the plasmid DNA.

### Phosphorylation of GAP45 with CDPK1

Phosphorylation of recombinant GAP45 and its variants with CDPK1 and [γ^32^P]ATP was performed in a solution containing 20 mM Tris HCl pH 8.0, 20 mM MgCl_2_, 1 mM CaCl_2_, 0.1 mM ATP (containing 0.1 MBq of [γ^32^P]), 100 nM CDPK1 and 8 µM GAP45 at 30°C for 10 min unless otherwise stated. Radioactivity incorporation was determined either by autoradiography or scintillation counting. For autoradiography, the assay was stopped by adding equal volumes of SDS-PAGE sample buffer and incubation at 90°C for 5 min, before subjecting samples to SDS-PAGE. The gel was fixed, dried and exposed to Kodak Biomax MR film to visualize radiolabelled protein bands. Band intensities were measured and analysed by ImageJ software (www.rsbweb.nih.gov/ij/). For scintillation counting, reactions were started at 5 to 10 min intervals to ensure identical incubation times and then spotted onto P81 phosphocellulose discs (Whatman) and immersed in 75 mM phosphoric acid for 5 min. The discs were rinsed with acetone, dried, and then transferred to scintillation vials to measure the radioactivity by Cherenkov counting using a scintillation counter [29].

### Subcellular fractionation, protein extraction from parasites and co-immunoprecipitation assay

Synchronized parasite populations were harvested and lysed with a hypotonic solution (10 mM Tris HCl, pH 8.0; 5 mM EDTA; and 1× protease inhibitor cocktail) for 1 h at 4°C. The mixture was centrifuged at 100,000 g for 10 min at 4°C and the supernatant (‘hypotonic lysis soluble fraction’) removed. The pellet was washed once with the hypotonic buffer and re- pelleted, prior to extraction with a high salt buffer (50 mM Tris HCl, pH 8.0; 5 mM EDTA; 500 mM NaCl; and 1× protease inhibitor cocktail) for 1 h at 4°C. The suspension was recentrifuged at 100,000 g and the supernatant (‘high salt soluble’) retained. The pellet was extracted again with 0.1 M sodium carbonate, pH 11.0 and refractionated to provide ‘carbonate-soluble’ and ‘carbonate-insoluble’ fractions.

For co-immunoprecipitation assays, purified parasites were lysed in 200 µl buffer (10 mM Tris-HCl, pH 7.5; 150 mM NaCl; 0.5 mM EDTA; 0.5% Nonidet P-40 [NP-40]; 1 mM PMSF and 1× protease inhibitor cocktail) on ice for 30 min. A soluble cell lysate was obtained by centrifugation at 20,000 g for 10 min at 4°C and diluted to 1000 µl with the same buffer lacking NP-40. GFP-Trap® beads (Chromotek) equilibrated with dilution buffer were added to the cell lysate for 1 h at RT, then recovered by centrifugation at 2000 g for 2 min at 4°C. The supernatant was retained and the beads were washed twice with the dilution buffer, then both fractions were mixed with SDS-PAGE sample buffer. After 10 min at 95°C soluble proteins were subjected to SDS-PAGE.

Protein samples were separated with 12% Nu-PAGE pre-cast Bis-Tris gels. Proteins were visualized by staining gels with Coomassie brilliant blue R-250, or subjected to western blotting. Following protein transfer to nitrocellulose membrane, the membrane was incubated in blocking solution (1% BSA in PBS containing 0.2% Tween 20 [PBST]) for 1 h and then incubated in primary antibody specific for GFP (Roche), GAP45, GAP50, MyoA, MTIP, SERA5, CDPK1 or MSP7. Following further washing, the membranes were incubated with secondary antibody (goat anti rabbit IgG or goat anti mouse IgG conjugated with horse radish peroxidase (HRP) (Biorad), as appropriate) and antibody binding detected by enhanced chemiluminescence reagent (ECL, GE Healthcare) and exposure to Biomax MR film (Kodak).

## Supporting Information

Figure S1 **The location of endogenous GAP45 and GAP45-GFP variants in developing schizonts.** (A) Staining of endogenous GAP45 protein using specific antibodies in 3D7 parasites 30, 33, 36, 39 and 42 hours post-invasion. (B) Dual antibody immunofluorescence of young (i) and mature (ii) schizonts using anti-GAP45 and anti-GFP antibodies. In young schizonts, the signal from GFP and GAP45 coincides perfectly for FL-GAP45 and C-GAP45 proteins, but not for N-GAP45. In mature schizonts, the staining of all of the GAP45 variants localises to the periphery of merozoites, but it is clear that for N-GAP45 there are many areas where this does not coincide with endogenous GAP45. For FL-GAP45 and C-GAP45 proteins, the colocalisation is exact. Scale bar is 2 µm.
(TIF)

Figure S2 **The location of GAP45-GFP and mutants (S89A, S103A, S89A/S103A, S89D, S103D, S89D/S103D) determined in (A) early and (B) late schizonts, by live fluorescence microscopy (green).** Parasite DNA was stained with Hoechst dye (blue); merged images and the differential interference contrast pictures are also shown. Scale bar is 2 µm.
(TIF)

Table S1 **In vitro CDPK1 phosphorylation of recombinant PfGAP45 and its variants.** The intensity of the band (autoradiography) was standardised against the corresponding protein concentration profile (coomassie) and analysed using ImageJ software. The data are presented as a mean percentage ± S.D.
(TIF)
