# Supplementary figures and images for: Subcellular Location, Phosphorylation and Assembly into the Motor Complex of GAP45 during Plasmodium falciparum Schizont Development
Source: PLoS One. 2012 Mar 30;7(3):e33845. doi: 10.1371/journal.pone.0033845 (PMC3316498; doi:10.1371/journal.pone.0033845)

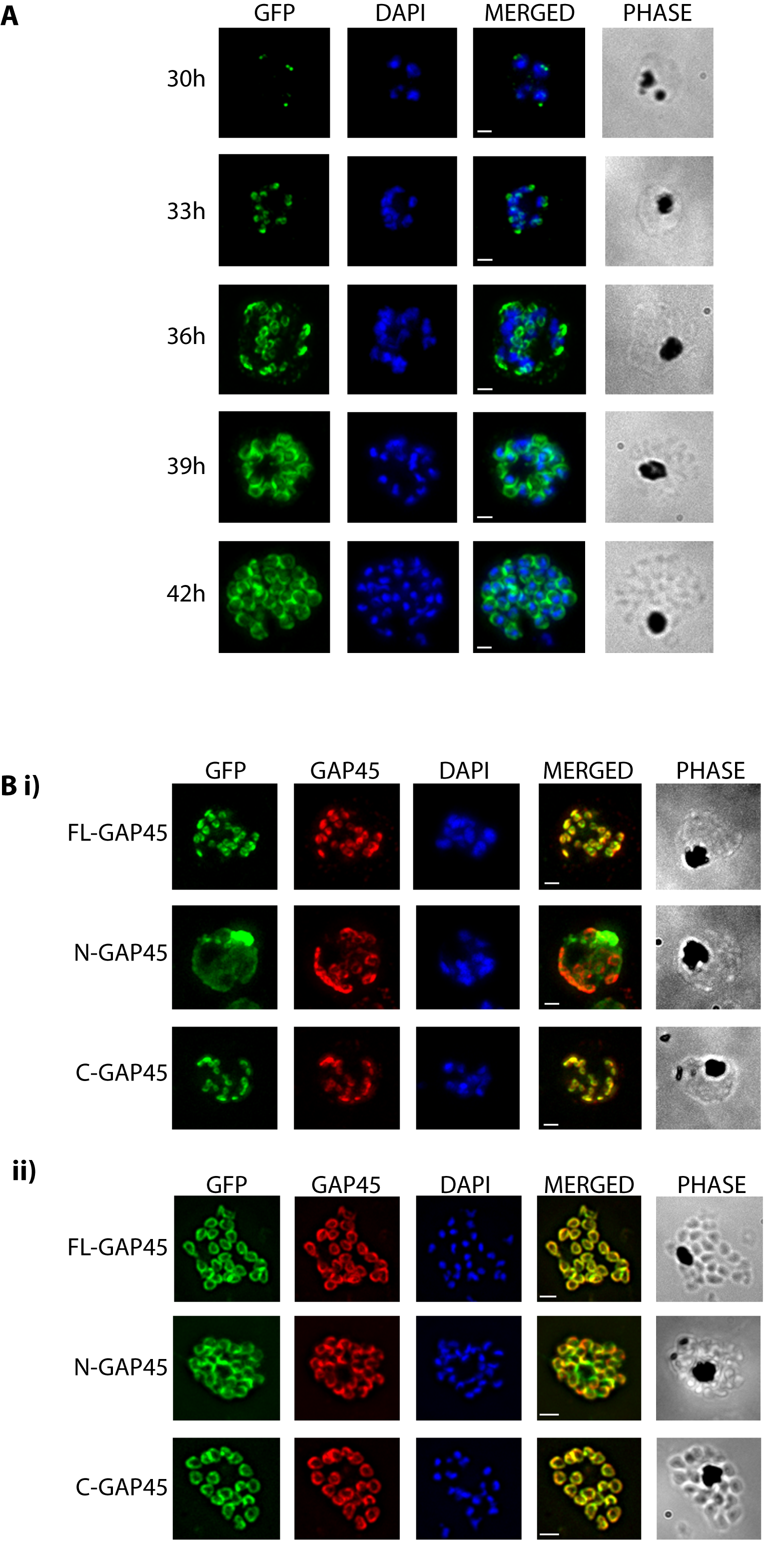

Supplement: Figure S1 — The location of endogenous GAP45 and GAP45-GFP variants in developing schizonts. (A) Staining of endogenous GAP45 protein using specific antibodies in 3D7 parasites 30, 33, 36, 39 and 42 hours post-invasion. (B) Dual antibody immunofluorescence of young (i) and mature (ii) schizonts using anti-GAP45 and anti-GFP antibodies. In young schizonts, the signal from GFP and GAP45 coincides perfectly for FL-GAP45 and C-GAP45 proteins, but not for N-GAP45. In mature schizonts, the staining of all of the GAP45 variants localises to the periphery of merozoites, but it is clear that for N-GAP45 there are many areas where this does not coincide with endogenous GAP45. For FL-GAP45 and C-GAP45 proteins, the colocalisation is exact. Scale bar is 2 µm. (TIF) [file pone.0033845.s001.tif]

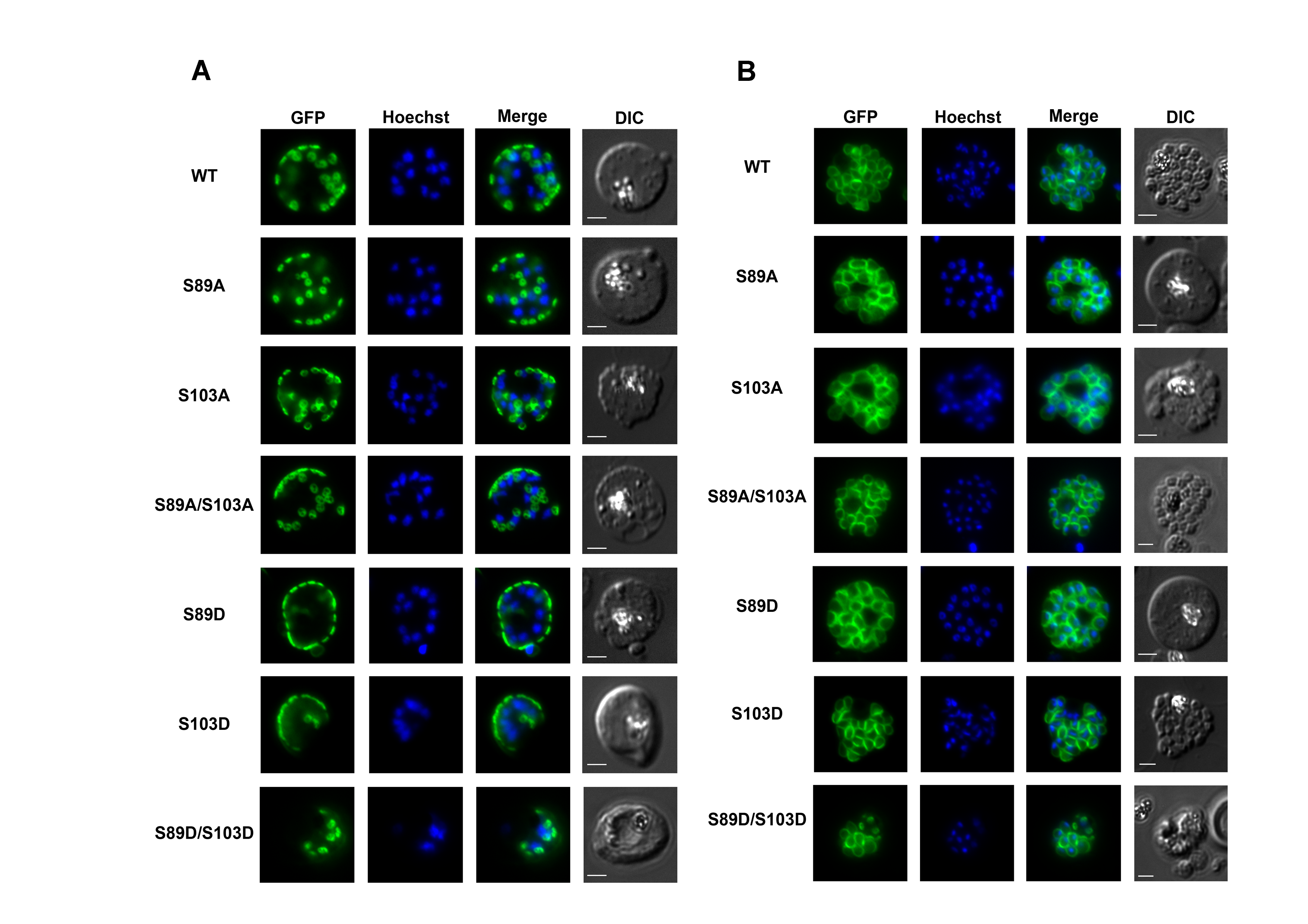

Supplement: Figure S2 — The location of GAP45-GFP and mutants (S89A, S103A, S89A/S103A, S89D, S103D, S89D/S103D) determined in (A) early and (B) late schizonts, by live fluorescence microscopy (green). Parasite DNA was stained with Hoechst dye (blue); merged images and the differential interference contrast pictures are also shown. Scale bar is 2 µm. (TIF) [file pone.0033845.s002.tif]

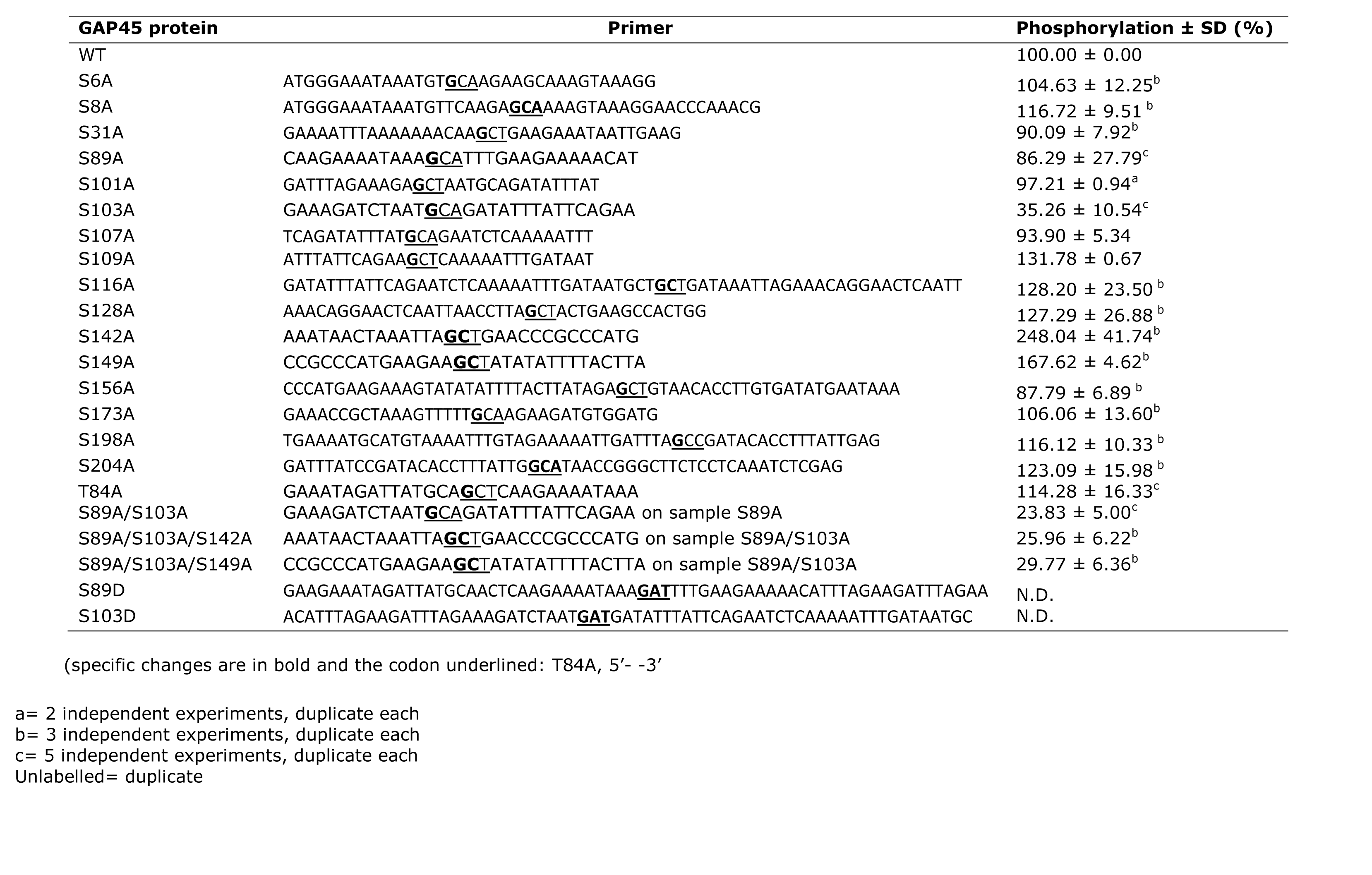

Supplement: Table S1 — In vitro CDPK1 phosphorylation of recombinant PfGAP45 and its variants. The intensity of the band (autoradiography) was standardised against the corresponding protein concentration profile (coomassie) and analysed using ImageJ software. The data are presented as a mean percentage ± S.D. (TIF) [file pone.0033845.s003.tif]
